# ^18^F-FDG PET-CT versus MRI for detection of skeletal metastasis in Ewing sarcoma

**DOI:** 10.1007/s00256-019-03192-2

**Published:** 2019-04-23

**Authors:** S. E. Bosma, D. Vriens, H. Gelderblom, M. A. J. van de Sande, P. D. S. Dijkstra, J. L. Bloem

**Affiliations:** 1 Department of Orthopedics, Leiden University Medical Center, Albinusdreef 2, 2333 ZA Leiden, The Netherlands; 2 Department of Radiology, Leiden University Medical Center, Leiden, The Netherlands; 3 Department of Medical Oncology, Leiden University Medical Center, Leiden, The Netherlands

**Keywords:** Ewing sarcoma, Diagnosis, Osseous metastasis, Positron emission tomography with computerized tomography, Magnetic resonance imaging

## Abstract

**Objective:**

To determine the level of discrepancy between magnetic resonance imaging (MRI) and ^18^F-FDG PET-CT in detecting osseous metastases in patients with Ewing sarcoma.

**Methods:**

Twenty patients with histopathologically confirmed Ewing sarcoma between 2000 and 2017 who underwent ^18^F-FDG PET-CT and MRI within a 4-week range were included. Each imaging modality was evaluated by a separate observer. Reference diagnosis of each lesion was based on histopathology or consensus of an expert panel using all available data, including at least 6 months’ follow-up. Sensitivity, specificity, and predictive values were determined. Osseous lesions were analyzed on a patient and a lesion basis. Factors possibly related to false-negative findings were evaluated using Pearson’s Chi-squared or Fisher’s exact test.

**Results:**

A total of 112 osseous lesions were diagnosed in 13 patients, 107 malignant and 5 benign. Seven patients showed no metastases on either ^18^F-FDG PET-CT or MRI. Forty-one skeletal metastases (39%) detected with MRI did not show increased ^18^F-FDG uptake on ^18^F-FDG PET-CT (false-negative). Lesion-based sensitivities and specificities were 62% (95%CI 52–71%) and 100% (48–100%) for ^18^F-FDG PET-CT; and 99% (97–100%) and 100% (48–100%) for MRI respectively. Bone lesions were more likely to be false-negative on ^18^F-FDG PET-CT if hematopoietic bone marrow extension was widespread and active (*p* = 0.001), during or after (neo)-adjuvant treatment (*p* = 0.001) or when the lesion was smaller than 10 mm (*p* < 0.001).

**Conclusion:**

Although no definite conclusions can be drawn from this small retrospective study, it shows that caution is needed when using ^18^F-FDG PET-CT for diagnosing skeletal metastases in Ewing sarcoma. Poor contrast between metastases and active hematopoietic bone marrow, chemotherapeutic treatment, and/or small size significantly decrease the diagnostic yield of ^18^F-FDG PET-CT, but not of MRI.

## Introduction

Ewing sarcoma is an aggressive primary bone sarcoma, predominantly affecting children and young adults [1, 2]. At the time of diagnosis, 20–25% of the patients present with pulmonary (70–80%) and/or osseous (40–50%) metastases. A multimodal approach to treatment drastically improved survival. In non-metastatic Ewing sarcoma 10-year overall survival is currently 55–65%, but survival in metastatic Ewing sarcoma is still dismal, with a 5-year overall survival of only 20–35% [3–5]. Principles of treatment consist of neo-adjuvant chemotherapy followed by local control of the primary tumor, by surgery, radiotherapy or both, and adjuvant chemotherapy [2, 4]. Detection of all metastatic lesions in patients with oligometastatic disease has become relevant, as a curative rather than a palliative treatment objective aimed at achieving local control at these sites has been reported to improve clinical outcome [6].

Pre-treatment imaging of newly diagnosed patients with Ewing sarcoma includes local staging with magnetic resonance imaging (MRI) and chest computerized tomography (CT) to detect pulmonary metastases [7]. Bone marrow biopsies and bone scintigraphy have been used to detect or exclude skeletal metastases. More recently 2-[^18^F]fluoro-2-deoxy-D-glucose positron emission tomography with CT (^18^F-FDG PET-CT) and whole-body MRI have been proposed to replace bone scintigraphy, because of higher sensitivity, and thus negative predictive value, to exclude skeletal metastasis [8–13]. With ^18^F-FDG PET-CT reflecting glucose metabolism of the lesions and MRI revealing morphological characteristics of metastatic deposits, these two screening techniques display different properties of the cancerous lesions: either functional or anatomical. No published literature directly comparing ^18^F-FDG PET-CT with whole-body MRI for the detection of skeletal metastases in Ewing sarcoma is currently available. Literature comparing the two modalities for skeletal metastases in other cancers shows conflicting results, with some researchers suggesting superiority for ^18^F-FDG PET-CT [11, 14, 15] and others for MRI [7, 8].

In our clinical practice we normally use both techniques. We frequently observed a mismatch between ^18^F-FDG PET-CT and MRI; in some patients, metastatic skeletal lesions detected by MRI were not detected with ^18^F-FDG PET-CT. Therefore, the purpose of this study was to retrospectively compare the diagnostic yield of ^18^F-FDG PET-CT with whole-body MRI for the detection of skeletal metastasis in Ewing sarcoma with a final diagnosis of an osseous lesion made by an expert panel using all follow-up data or histopathology (where available).

## Materials and methods

### Study design and patients

The local ethics board approved this retrospective study and waived the requirement for informed consent. We searched the database of our tertiary referral bone sarcoma center for all patients diagnosed with Ewing sarcoma between 1 January 2010 and 1 January 2018. Patients were eligible for inclusion when fulfilling all of the following criteria:

1. Histopathologically confirmed Ewing sarcoma
2. Treatment and diagnostic work-up according to the EUROpean Ewing tumor Working Initiative of National Groups–Ewing Tumor Studies (EURO-EWING) 2008 or 2012 protocol
3. Whole-body ^18^F-FDG PET-CT and whole-body or large field-of-view regional MRI performed within a 4-week range

All sets of scans performed at baseline were executed before the start of treatment. All sets of scans performed during follow-up were executed at the same treatment stage or moment during follow-up. In the case of multiple paired ^18^F-FDG PET-CT and MRI scans of a single patient, the first available set was used. We performed an additional analysis for therapy-naïve patients and patients who were already treated separately to check if this had an impact on detection.

**Fig. 1:**
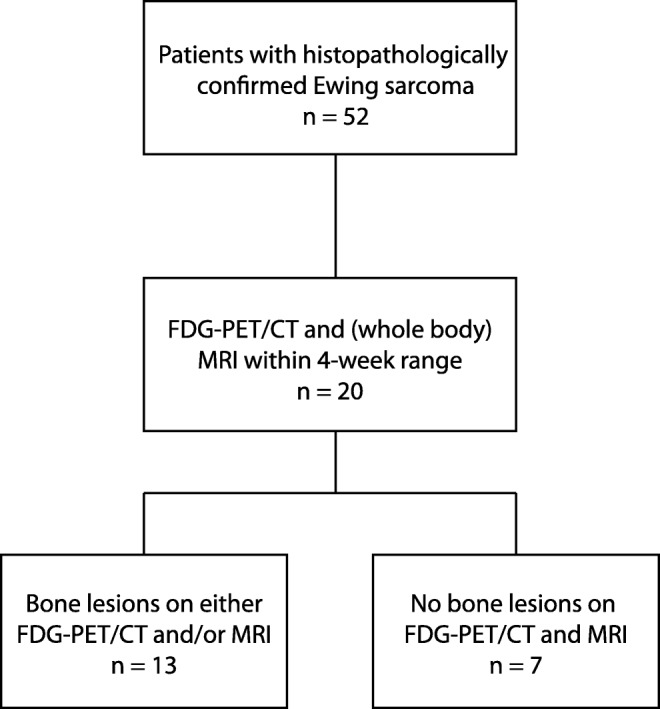
Flowchart of the inclusion process

We identified 52 patients with histopathologically confirmed Ewing sarcoma and included 20 patients who had undergone both ^18^F-FDG PET-CT and MRI, either at diagnosis or during follow-up, within a 4-week range. Figure 1 shows a flowchart of the inclusion process.

### ^18^F-FDG PET-CT acquisition and evaluation

After at least 6 h of fasting (sugar-free liquids were allowed) and validation of normoglycemia (<11.1 mmol/L), patients were intravenously injected with ^18^F-FDG (dose dependent on bodyweight, scanner sensitivity, and acquisition duration). After a ~60-min resting period, low-dose CT and PET images were acquired from vertex to toes on multiple PET-CT scanners (Siemens Biograph Horizon, Siemens Biograph mCT, and Philips Gemini TF) in our own and six referring centers according to the European Association of Nuclear Medicine (EANM) procedure guidelines for tumor imaging in FDG PET-CT (version 2.0) [16]. Analysis of ^18^F-FDG PET-CT images was repeated for all scans and was primarily done by visual assessment. The decision of the conspicuity of a skeletal lesion was determined by an experienced PET-CT reader (DV, nuclear medicine physician, 10 years’ experience), blinded to clinical and histopathological information and other imaging examinations. Visible lesions on ^18^F-FDG PET-CT were scored positive (i.e., suspected malignancy), negative (i.e., suspected benignity) or were inconclusive. Focal bone uptake visible in three orthogonal plans, higher than the surrounding bone marrow, without a clear benign cause (e.g., growth plate) was scored as suspected malignancy (positive). If additional imaging was suggested for confirmation, it was scored as “inconclusive.” All other lesions were scored benign (negative). Semiquantitative assessment of PET-positive lesions by measurement of their maximum standardized uptake value (SUV_max_) was performed and related to the SUV_max_ of the mediastinal blood pool and healthy right liver activity, resulting in the six-point scale presented in Table 1. Last, metabolically active hematopoietic bone marrow extension and activity were quantified using a visual four-point scale defined a priori based on the literature [17–19]. The visual four-point scale was defined as follows: 0—metabolically active hematopoietic bone marrow only present in the spine/pelvis without increased activity (SUV_max_ lower than or equal to the liver); 1—metabolically active hematopoietic bone marrow only present in the spine/pelvis with increased activity (SUV_max_ higher than the liver); 2—metabolically active hematopoietic bone marrow extension up to the pertrochanteric femoral and/or subcapital humeral regions with increased activity; 3—metabolically active hematopoietic bone marrow extension beyond the pertrochanteric femoral and/or subcapital humeral regions with increased activity. For analysis we dichotomized the data, normal hematopoietic bone marrow was defined by a score of 0 or 1 and widespread hematopoietic bone marrow extension and activity was defined as a score of 2 or 3.

**Table 1:** Semiquantitative assessment of lesion ^18^F-FDG uptake according to a six-point scale

Score	Description
0	No uptake
1	Notable uptake < mediastinal blood pool
2	Notable uptake > mediastinal blood pool, but < liver
3	Notable uptake ≈ liver uptake (±10%)
4	Intense uptake > liver, but ≤2,5× liver
5	Intense uptake >2.5× liver uptake

### MRI acquisition and evaluation

Whole-body MRI was performed in 14 patients using a 1.5-T system (Philips Healthcare, Best, the Netherlands). Standard protocol included T1-weighted turbo spin echo (TSE) with a slice thickness of 5 mm, repetition time (TR) of 727 ms, echo time (TE) of 15 ms, and short tau inversion recovery (STIR) sequences using four stations in the coronal plane with a slice thickness of 5 mm, TR 7,192 ms, TE 50 ms, inversion time 210 m, and sagittal T1 and STIR sequences of the entire spine using the above mentioned MRI parameters for T1 and STIR. In 6 patients a large field-of-view regional MRI using the same parameters was obtained. Additional sequences that were created in these regional scans were not reviewed for current analysis. In these 6 patients, only regions imaged by both modalities were evaluated and compared. In 1 of these 6 patients, ^18^F-FDG PET-CT showed three osseous lesions outside the MRI field of view, which were not included in the current analysis.

Magnetic resonance images were evaluated by one radiologist specialized in MRI imaging (JLB, >10 years’ experience), blinded to clinical and histopathological information and other imaging examinations. Malignancy on MRI was based on the assessment of morphological and signal characteristics. A nodule presenting with a lower signal than the surrounding bone marrow on T1 and a higher signal on STIR sequences was scored positive (i.e., suspected malignancy). All other lesions were considered benign (negative). Next, quantitative assessment of MRI-positive lesions was performed by measuring the size (defined as maximum diameter) of the lesion. Lesion measurement was dichotomized at the 10-mm diameter level.

### Reference method

Histopathological correlation for every osseous lesion depicted was not available in most of the lesions for ethical reasons: in only 2 patients was confirmation of skeletal metastasis by biopsy available. In the other 11 patients with osseous lesions on imaging, the final decision of the true status of the osseous lesion was made by consensus using an expert panel consisting of a board-certified radiologist and nuclear physician. All available clinical information, including therapy schedules, response to treatment, and follow-up imaging examinations (^18^F-FDG PET-CT, MRI, diagnostic CT) were used to reach the overall decision. Patients were routinely evaluated every 3 months by ^18^F-FDG PET-CT and/or MRI. The mean imaging follow-up was 15.7 months (range 1.8 to 31.3 months). Two patients died because of progressive disease shortly (1.8 and 3.8 months) after imaging was performed and no obduction was performed. In the other 18 patients (7 without osseous lesions and 11 with osseous lesions) at least 6 months of follow-up imaging examinations were available to determine the true status of a bone lesion.

Change in imaging characteristics, increase in size of the entire lesion or the extra-osseous component, or increased ^18^F-FDG uptake of the lesions indicated malignancy. Response to treatment was used as a sign of malignancy and was defined as a decrease in ^18^F-FDG-uptake, a decrease in size of the lesions, or complete disappearance of the lesion. A lesion was considered benign if a specific diagnosis could be made, if it showed no change over time, especially when other lesions changed in response to treatment, or if progressive disease was diagnosed in other sites of the skeleton.

### Data analysis

Each visible lesion was scored separately as being malignant, benign, or inconclusive on either imaging modality. Number of lesions and location were determined for both ^18^F-FDG PET-CT and MRI. Location was defined using 11 predefined skeletal body regions: skull; ribs; pelvis; cervical spine; thoracic spine; lumbar spine; proximal upper extremity; distal upper extremity; proximal lower extremity; distal lower extremity; other regions (scapula, sternum, clavicles). If a patient presented with multiple lesions in one region, a maximum of four lesions was included for analysis to avoid the bias of few patients with a very large number of lesions.

In the case of discordance between ^18^F-FDG PET-CT and MRI, we searched for potential causes in a separate consensus meeting by the expert panel, after all patients had been scored by the individual observers. Additionally, if osseous lesions showed no ^18^F-FDG uptake we evaluated whether these lesions were visible on the low-dose CT of the ^18^F-FDG PET-CT, using MRI as guidance.

### Statistical analysis

Both patient-based analysis and lesion-based analysis were performed and the results are described as true-positive, true-negative, false-positive, and false-negative. Lesions that were scored as inconclusive on imaging were considered positive for this analysis. Osseous lesions were also evaluated and reported as true-positive, false-positive, true-negative, and false-negative in patient-based and lesion-based analysis.

In the case of a discordant finding within a single patient, a true-positive lesion superseded all other lesions, including false-negative, true-negative, and false-positive lesions. Thus, if a subject presented with at least one true-positive lesion, that patient was considered true-positive for that imaging modality. In the absence of a true-positive lesion, a false-negative lesion superseded a true-negative or false-positive lesion. Therefore, if imaging was false-negative in at least one site, that patient was considered false-negative overall for that modality. Using this approach, we addressed the question whether or not recurrent/metastatic disease was present.

We computed accuracy, sensitivity, specificity, and positive and negative predictive values using classical equations. The 95% confidence intervals of these test characteristics were computed using the absolute Clopper–Pearson interval (using beta-distribution). We explored the following factors to be related to false-negative findings: lesion size, location, hematopoietic bone marrow extension, and treatment stage (before treatment, on treatment, recurrence after treatment) using Pearson’s Chi-squared or Fisher’s exact test, where appropriate.

## Results

### Patient population

In 7 of the 20 patients ^18^F-FDG PET-CT and MRI were both negative for the presence of osseous lesions. All these patients were routinely evaluated every 3 months using ^18^F-FDG PET-CT and MRI and none of these patients was diagnosed with skeletal metastasis within the next 6 months. All these cases were considered true-negative on both imaging modalities. Later 3 of these patients developed pulmonary and/or skeletal metastasis during long0term follow-up. At the termination of our study, the 4 other patients were alive with no evidence of disease and the 3 patients who later developed metastases died because of recurrent or progressive disease.

In the remaining 13 patients (Table 2), osseous lesions on any or both imaging modalities were reported to be present. A total of 112 bone lesions were identified using our standard of reference; 89 in the axial skeleton (30 vertebral, 15 rib, 33 pelvic, 4 glenoid, 1 acromion, 3 clavicles, 2 sternum, 1 skull), and 23 in the peripheral skeleton (16 lower extremities, 7 upper extremities). Four patients had already been treated at the time of imaging, whereas all imaging had been performed before the start of treatment in the other 9 patients. By the end of our study, 7 patients had died because of progressive disease, 6 patients were alive, 4 of whom were undergoing palliative treatment and 2 were alive with no evidence of disease.

**Table 2:** Patient-based and lesion-based diagnosis of bone lesions in patients with at least one abnormality

Number	Age/sex	Primary tumor	Purpose of the study	Standard of reference	Number of lesions^b^	^18^F-FDG PET-CT		MRI	
						PB	LB	PB	LB
1	23/male	Tibia	Follow-up^a^	CF	16	TP	6	TP	16
2	22/male	Femur	Follow-up	CF	2	TP	2	TP	2
3	23/male	Femur	Staging	CF	3	TP	3	TP	3
4	26/male	Pelvic	Staging	CF	25	TP	19	TP	24
5	17/male	Tibia	Follow-up	CF	16	TP	6	TP	16
6	17/female	Rib	Follow-up	CF	2	FP	2	TN	0
7	5/female	Tibia	Staging	HP	1	TP	1	TP	1
8	23/male	Humerus	Staging	CF	24	TP	19	TP	23
9	8/female	Tibia	Staging	HP	2	TP	1	TP	2
10	23/male	Rib	Staging	CF	11	TP	4	TP	11
11	22/male	Pelvic	Staging	CF	5	TP	3	TP	5
12	16/male	Fibula	Staging	CF	1	TP	1	TP	1
13	29/female	Femur	Staging	CF	2	TP	2	TP	2
Total					112		69		106

*CF* clinical follow-up, *FN* false-negative, *FP* false-positive, *HP* histopathology, *LB* lesion-based, *PB* patient-based, *TP* true-positive, *TN* true-negative

^a^Active chemotherapy

^b^On either of the imaging modalities

### Patient-based analysis for PET-CT vs MRI

Twelve out of 13 patients (92.3%) with suspected skeletal metastasis on either of the two imaging modalities were correctly identified by ^18^F-FDG PET-CT and MRI concordantly, and thus were considered true-positive.

**Fig. 2:**
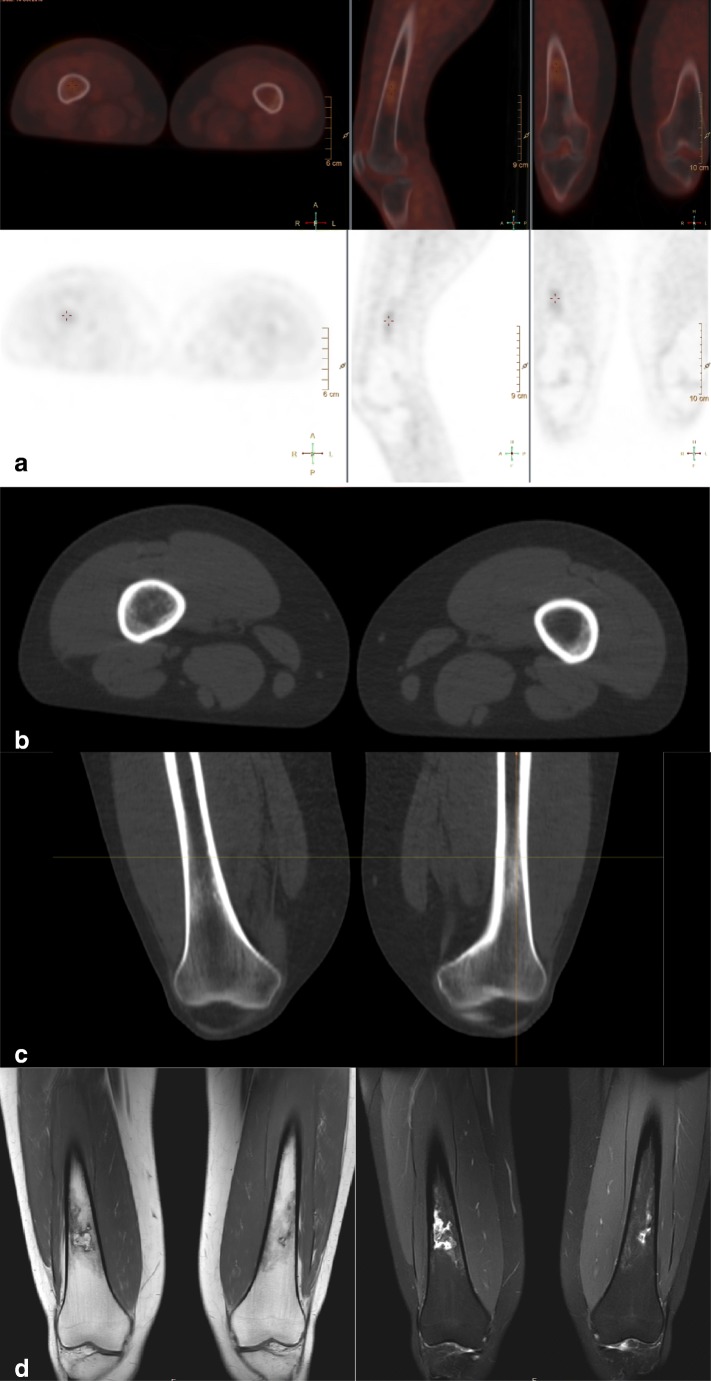
False-positive lesions on ^18^F-FDG PET-CT. A 19-year-old woman diagnosed with localized Ewing sarcoma of the seventh rib. Six months after initial treatment consisting of six courses of vincristine, ifosfamide, doxorubicin, and etoposide (VIDE) chemotherapy, 8 courses of vincristine, actinomycin-D, and ifosfamide (VAI) chemotherapy, radiation therapy and surgery, imaging was performed because of chest pain, with local recurrence suspected. **a**^18^F-FDG PET-CT showed two lesions with ^18^FDG-uptake in both femora of which the true nature could not be clearly defined; based on the information available they were classified as inconclusive (positive). **b** and **c** Low-dose CT images in the transverse and coronal planes of the suspected bone lesions showing sclerosis. **d** MRI T1- and T2-weighted images show bilateral bone infarctions and no sign of malignancy

In 1 patient (7.7%) ^18^F-FDG PET-CT showed two almost symmetrical lesions with subtle sclerosis and ^18^F-FDG-uptake in both distal femoral diaphyses of which the true nature could not be clearly defined. Based on the information available, these lesions were classified as inconclusive. On MRI and CT, a diagnosis of bilateral bone infarctions was made, as confirmed by the expert panel (Fig. 2). During follow-up, the patient presented with progressive disease, and died 16.3 months later. No metastatic lesions developed at the distal femora during the disease progression and the bone infarctions did not change; the ^18^F-FDG PET-CT was therefore considered false-positive. There were no false-positive MRI and no false-negative scans.

The positive predictive values (PPVs) with corresponding 95% confidence interval (95%CI) of ^18^F-FDG PET-CT and MRI were therefore 92% (62–100%) and 100% (72–100%) respectively. The sensitivities were 100% (72–100%) for ^18^F-FDG PET-CT and 100% (72–100%) for MRI.

### Lesion-based analysis

A total of 112 lesions in 13 patients were identified and characterized as malignant or benign by the expert panel using the predefined standard of reference. Of these, 107 lesions (95.5%), present in 12 patients, were considered malignant. Five lesions in 4 patients were considered to be benign.

The data from the lesion-based analysis are presented in Table 3. ^18^F-FDG PET-CT and MRI were concordantly positive in 65 osseous lesions (58%), whereas 41 osseous lesions (37%) in 7 patients were observed on MRI only, compared with 4 osseous lesions (4%) in 3 patients observed on ^18^F-FDG PET-CT only. Two osseous lesions (1%) in 1 patient were defined as being benign on both imaging modalities.

**Table 3:** Lesion-based analysis of all osseous lesions

		PET		
		+	–	Total
MRI	+	65	41	106
	–	4	2	6
	Total	69	43	112

**Fig. 3:**
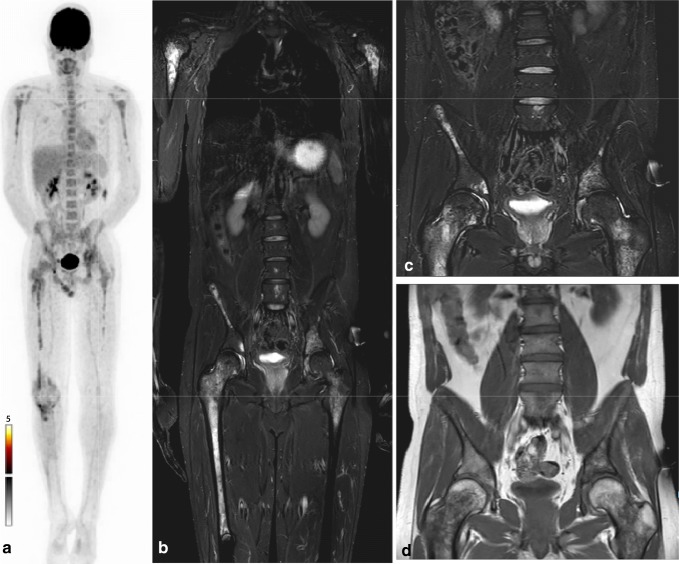
False-negative lesions on ^18^F-FDG PET-CT with widespread hematopoietic bone marrow activity. A 23-year-old man diagnosed with localized Ewing sarcoma of the right proximal tibia. Images obtained 6 months after initial treatment (6 × VIDE, surgery, 8 × VAI), at this time undergoing second-line chemotherapy because of recent distant metastasis. **a**^18^F-FDG PET-CT with symmetrical ^18^F-FDG uptake in the axial skeleton and proximal extremities. This was classified benign (negative) owing to anemia or recent chemotherapy. **b** T1-weighted short tau inversion recovery (STIR) MRI images with multifocal metastatic lesions throughout the whole axial skeleton. **c** STIR images with several skeletal metastases in the left and right ilium and fifth lumbar vertebral body. **d** T1-weighted turbo spin echo (TSE) images with several skeletal metastases in the left and right ilium and fifth lumbar vertebral body

**Fig. 4:**
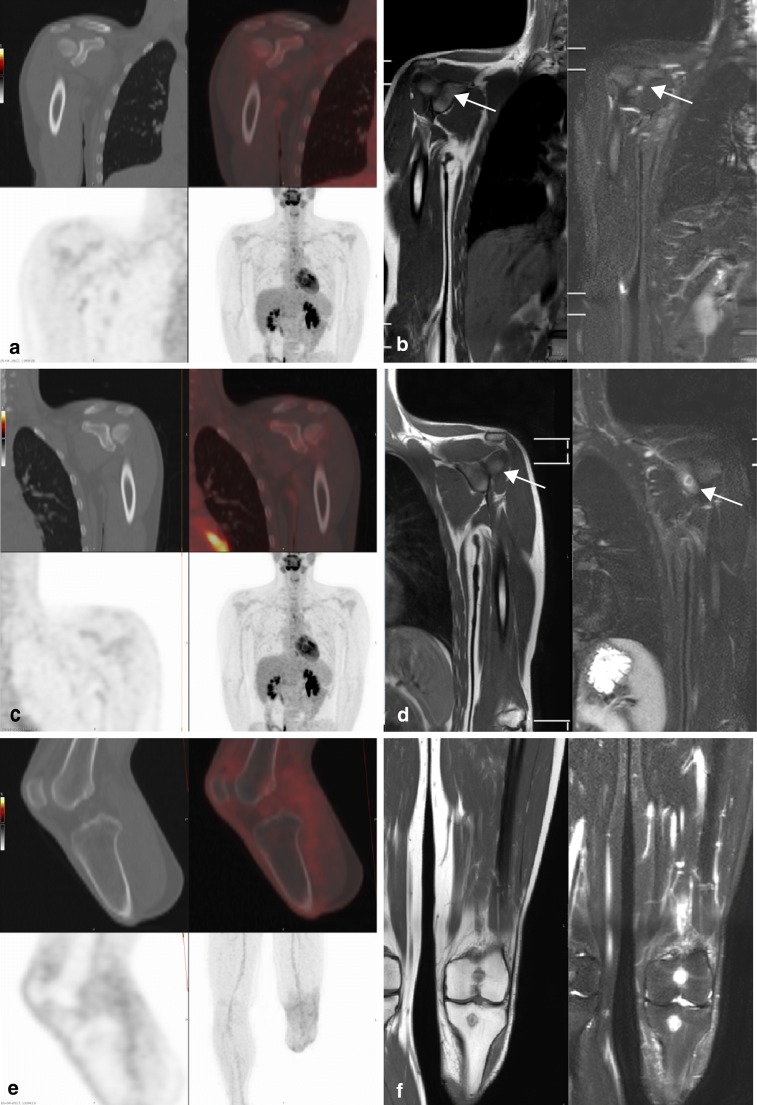
False-negative lesions on ^18^F-FDG PET-CT. A 17-year-old boy diagnosed with localized Ewing sarcoma of the distal tibia. Images obtained 1 year after finishing treatment (6 × VIDE, amputation, 8 × VAI). **a**^18^F-FDG PET-CT showing no increased ^18^F-FDG-uptake at the glenoid of the right shoulder. **b** T1-weighted (*left*) and STIR (*right*) images showing a small nodule (*arrow*) with a high degree of suspicion for metastasis at the glenoid of the right shoulder. **c**^18^F-FDG PET-CT showing no increased ^18^F-FDG-uptake or lytic changes on low-dose CT at the glenoid of the left shoulder. **d** T1-weighted (*left*) and STIR (*right*) images showing a nodule (*arrow*) with a high degree of suspicion for metastasis at the glenoid of the left shoulder. **e**^18^F-FDG PET-CT showing no increased ^18^F-FDG-uptake or lytic changes on low-dose CT at the left proximal tibia and distal femur. **f** T1-weighted (*left*) and STIR (*right*) images showing two nodules with a high degree of suspicion for metastasis at the left proximal tibia and distal femur

**Fig. 5:**
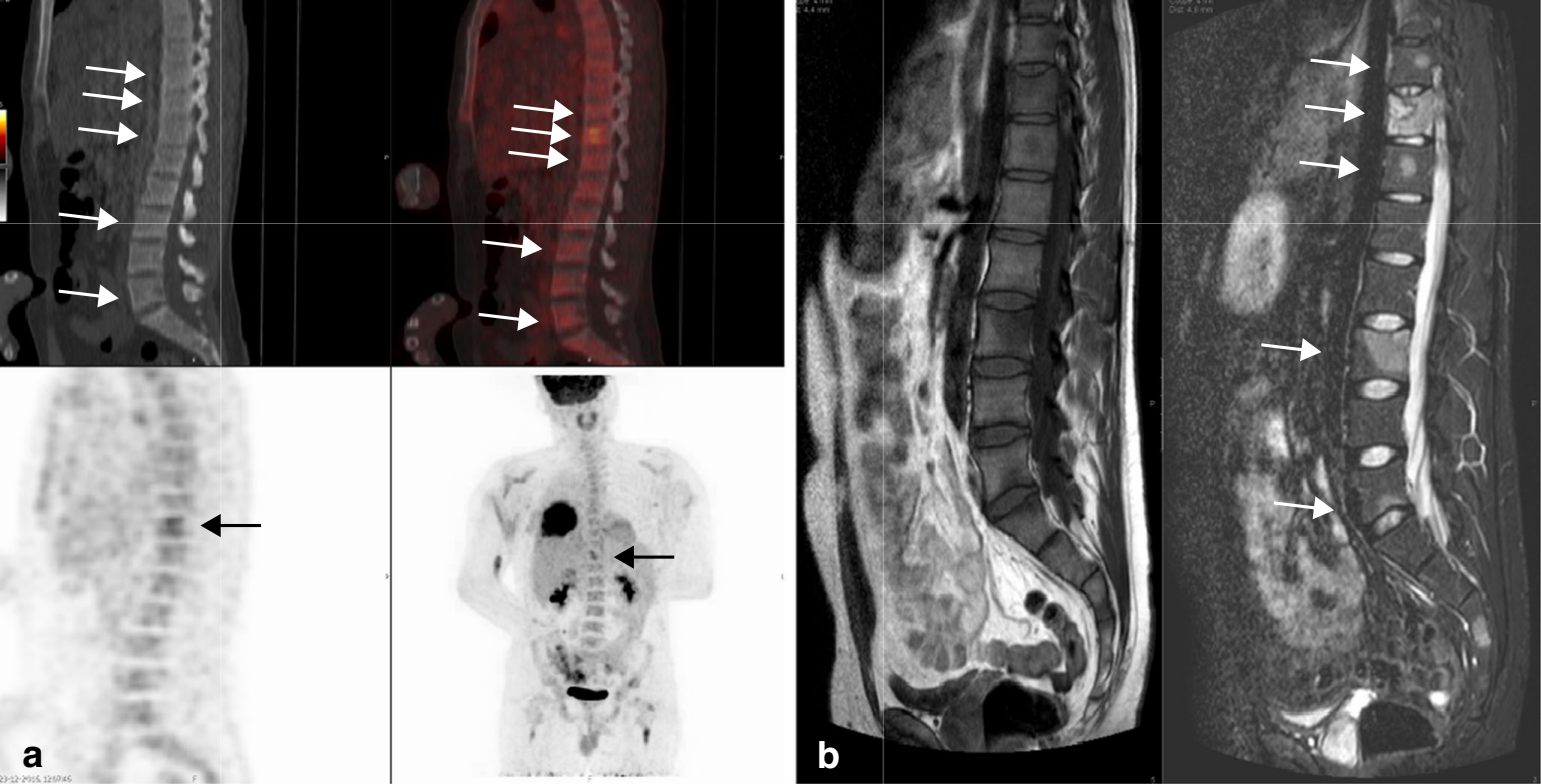
False-negative lesions on ^18^F-FDG PET-CT (*arrows*). A 23-year-old man presenting with metastatic Ewing sarcoma of the right seventh rib. Images obtained at diagnosis, before the start of treatment. **a**^18^F-FDG PET-CT showing increased ^18^F-FDG-uptake at the eleventh thoracic vertebrae only. **b** T1-weighted (*left*) and STIR (*right*) images showing nodules with a high degree of suspicion for metastasis at the tenth, eleventh, and twelfth thoracic vertebrae and the third and fifth lumbar vertebrae

The 41 lesions visible on MRI only were all considered to be malignant according to the standard of reference and therefore true-positive. These 41 lesions were thus false-negative on ^18^F-FDG PET-CT. Most of these 41 lesions (36 lesions; 88%) was located in the axial skeleton: spine (20 lesions; 49%), rib (2 lesions; 5%), pelvis (7 lesions; 17%), other axial regions (glenoid and clavicles; 7 lesions, 17%). Only 5 lesions (12%) were found in the extremities. Lesions were not more likely to be false-negative on ^18^F-FDG PET-CT when located in the axial skeleton compared with an extremity location (40% versus 30%; *p* = 0.522). In addition to location in the axial skeleton, we evaluated possible confounders, potentially explaining the false-negative lesions on ^18^F-FDG PET-CT. In the 9 therapy-naïve patients, lesions were less likely to be false-negative on ^18^F-FDG PET-CT compared with the 4 patients who had already started treatment (26% versus 58%; *p* = 0.001). In 3 patients with false-negative lesions on ^18^F-FDG PET-CT, widespread hematopoietic bone marrow extension and activity were present. Lesions were more likely to be false-negative on ^18^F-FDG PET-CT when widespread active red bone marrow was present (55% versus 22%; *p* = 0.001). In 1 patient, recent chemotherapy led to bone marrow rebound on ^18^F-FDG PET-CT obscuring 10 lesions, all located in the axial skeleton (Fig. 3). Ten lesions in 5 patients were smaller than 10 mm and all but one of these lesions were located in the axial skeleton. Lesion size below 10 mm led to more false-negative lesions on ^18^F-FDG PET-CT (100% versus 30%; *p* < 0.001). Figures 4 and 5 provide examples of the false-negative lesions on ^18^F-FDG PET-CT.

Of these 41 false-negative lesions on ^18^F-FDG PET-CT, 39 could not be identified on the low-dose CT of the ^18^F-FDG PET-CT by the expert panel. The expert panel identified two skeletal metastases present in 2 patients that were visible on the low-dose CT as small osteolytic lesions, positive on MRI, but interpreted as negative on ^18^F-FDG PET-CT. One of these two false-negative lesions was in close proximity to the physiologically ^18^F-FDG-positive growth plate and (thus) falsely interpreted as negative. The other false-negative lesion was located at the posterior iliac crest and was interpreted as reactive uptake owing to bone-marrow biopsy because of its location. However, no bone marrow biopsy had been performed, and the small lytic lesion on low-dose CT had been interpreted as iatrogenic. All other 39 false-negative lesions showed no ^18^F-FDG uptake on PET-CT. On MRI and during imaging follow-up, this lesion was classified as malignant and thus interpreted as false-negative by ^18^F-FDG PET-CT.

Three out of four bone lesions visible on ^18^F-FDG PET-CT only were considered false-positive. These included two lesions diagnosed as bone infarctions in a single patient (Fig. 1) and a bone lesion in the eighth thoracic vertebral body. During imaging follow-up of over 1 year, no change in the lesion in the eighth thoracic vertebral body was seen. A diagnosis could not be made, however, as no progression or change of the lesion had been seen in over 1 year; while the patient was having progressive disease under treatment, the lesion was regarded as benign according to our reference standard and therefore as false-positive on ^18^F-FDG PET-CT.

The one PET-positive lesion that was false-negative on MRI according to the standard of reference was missed owing to partial volume effects. This small lesion (<1 cm) fell between two slices because of the slice gap of 10% with a slice thickness of 5 mm.

Table 4 provides an overview of the lesion-based analysis relative to the standard of reference for each imaging modality separately. The lesion-based PPV for ^18^F-FDG PET-CT and MRI were 96% (95%CI 91–100%) and 100% (97–100%) respectively. The lesion-based NPV for ^18^F-FDG PET-CT and MRI were 5% (0–11%) and 83% (54–100%) respectively. Sensitivities and specificities for these modalities were 62% (95%CI 52–71%) and 100% (95%CI 48–100%) for ^18^F-FDG PET-CT and 99% (97–100%) and 100% (48–100%) for MRI respectively. Accuracy was 63% (95%CI 54–72%) for ^18^F-FDG PET-CT and 99% (95%CI 95–100%) for MRI.

**Table 4:** Lesion-based analysis according to the standard of reference

		Standard of reference	
		Malignant	Benign
PET	+	66	0
	–	41	2
	Indeterminate	0	3
MRI	+	106	0
	–	1	5

### Semiquantitative assessment of ^18^F-FDG PET-CT

Most of the true-positive PET lesions (67 out of 107, 63%) had a score of 3 (notable uptake with a SUV_max_ of ±10% compared with the liver uptake) or higher. The remaining 40 lesions showed no visible uptake on ^18^F-FDG PET-CT or only showed low uptake and were considered benign (SUV_max_ lower than the blood pool; Table 5).

**Table 5:** Scores of ^18^F-FDG PET-CT lesions

		Total	Score					
			0	1	2	3	4	5
Standard of reference	Malignant	107	36	2	2	3	52	12
	Benign	5	0	0	2	0	3	0
PET-CT interpretation	Positive/indeterminate	69	0	0	4	2	53	10
	Negative	43	36	2	0	1	2	2

Score based on maximum SUV_max_ divided into standard of reference and visual ^18^F-FDG PET-CT interpretation

## Discussion

Accurate detection and localization of all metastases in oligometastatic Ewing sarcoma are clinically relevant as metastasectomy or radiation of these sites potentially provides a curative approach [6].

Of all detected lesions, 95.5% were considered malignant by our reference standard. The PPVs of both ^18^F-FDG PET-CT and MRI are high and the number of false-positive lesions low. Thus, in this young patient population, any lesion should be considered malignant until proven otherwise. In 39% of confirmed metastases detected with MRI no increased ^18^F-FDG uptake was present and these were thus missed on ^18^F-FDG PET-CT. Only 2 (5%) of these ^18^F-FDG negative metastases could retrospectively be found by the expert panel on the low-dose CT images. On a patient basis, ^18^F-FDG PET-CT and MRI both performed well. In only 1 patient without skeletal metastasis, PET-CT showed inconclusive and thus, according to predefined criteria, false-positive findings, whereas MRI was true-negative. All other patients with suspected skeletal metastasis were correctly identified by both imaging modalities.

Our results cannot be compared with the existing literature, as published reports on the performance of MRI relative to ^18^F-FDG PET-CT are normally based on inclusion of heterogeneous populations with different types of malignancy. In general, ^18^F-FDG PET-CT and MRI perform well, but there is no consensus in the literature about differences in performance in specific tumor types such as Ewing sarcoma.

The question is what can explain the difference between ^18^F-FDG PET-CT, which is based on the glucose metabolism within the tumor, and MRI, which is based on the morphology of the metastases in bone marrow. It seems that there are at least three factors that, in combination, cause these false-negatives: the activity of normal bone marrow on ^18^F-FDG PET-CT, small lesion size, and variation in glucose consumption.

First, the presence of hematopoietic bone marrow has a significant impact on the performance of ^18^F-FDG PET-CT, as it decreases the contrast between normal and abnormal ^18^F-FDG uptake. Patients with Ewing sarcoma are young and have active hematopoietic bone marrow in the axial skeleton [20]. Also, anemia, previous treatment with chemotherapy or medication may lead to increased activity of hematopoietic marrow in Ewing sarcoma patients. ^18^F-FDG has an increased uptake in hematopoietic marrow relative to yellow bone marrow, thereby increasing the background activity on ^18^F-FDG PET-CT. As hematopoietic bone marrow is typically located in the axial skeleton and proximal extremities, it is no surprise that most false-negative lesions (88%) were located in the axial skeleton.

Second, lesion size also contributes to the large number of false-negative lesions on ^18^F-FDG PET-CT. Ten out of 41 false-negative lesions (24%) were smaller than 10 mm and these smaller lesions were more likely to be false-negative on ^18^F-FDG PET-CT.

Last, changes in the tumor micro-environment of Ewing sarcoma that affect the glucose metabolism may also contribute to the large amount of false-negative lesions of ^18^F-FDG PET-CT [21].

This study has a few limitations. First, Ewing sarcoma is a rare disease; therefore, numbers are low. In addition, we performed a retrospective study; thus, selection bias may play a role in that there could have been a reason for the second imaging modality to be performed after the first one (i.e., no independence). Second, histopathological confirmation was not available in the largest proportion of the lesions. Follow-up imaging was used as a reference method in most of the lesions. Although this is an accepted tool for lesion characterization, it could affect the accuracy of our results. Third, imaging analysis was performed by one experienced nuclear medicine physician and one experienced radiologist. In general, ^18^F-FDG PET-CT is evaluated by a nuclear medicine physician and MRI by a musculoskeletal (MSK) radiologist. The large number of radiologists allows for more specialization. If two MSK radiologists had evaluated all imaging data, two false-positive lesions (the two bone infarctions in one patient, Fig. 1) and two false-negative lesions (that could in retrospect be found on low-dose CT) might have been prevented and could thus be considered as an interpretation error. None of the other lesions showed ^18^F-FDG-uptake and were not visible on low-dose CT. Last, in 6 out of 20 cases, no whole-body MRI was available for comparison and the specificity of the two techniques could therefore not be determined. However, only three osseous lesions visible on ^18^F-FDG PET-CT were not imaged by MRI.

In conclusion, although no definite conclusions can be drawn from this small retrospective study, we conclude that caution is needed when using ^18^F-FDG PET-CT for diagnosing skeletal metastases in Ewing sarcoma, as 39% of metastases in this cohort seen on MRI are not detected with ^18^F-FDG PET-CT. Suggestions for the main causes are poor contrast between metastases and active hematopoietic bone marrow, small size, and potentially changes in glucose metabolism in metastases of Ewing sarcoma. Further research is needed to evaluate the discrepancy in ^18^F-FDG PET-CT and MRI findings and to confirm our results.
